# *QuickStats:* Number of Natural Heat-Related Deaths,* by Sex and Age Group — National Vital Statistics System, United States, 2018

**DOI:** 10.15585/mmwr.mm6930a6

**Published:** 2020-07-31

**Figure:**
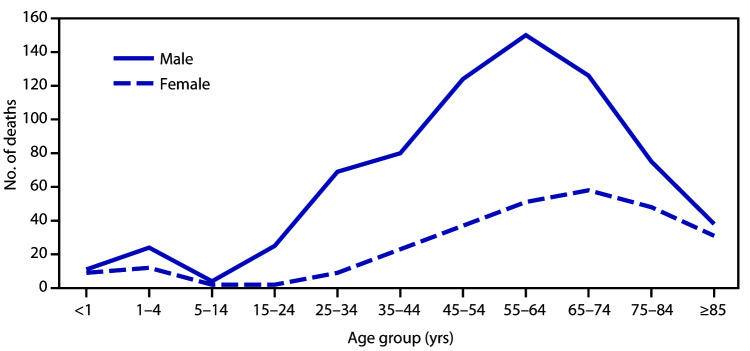
In 2018, natural heat exposure was associated with 726 deaths among males and 282 deaths among females. Among males, the highest number of heat-related deaths was for those aged 55–64 years (150) and among females for those aged 65–74 years (58). The lowest numbers were for males (four) and females (two) aged 5–14 years. Approximately 72% of heat-related deaths were among males.

**Source:** National Vital Statistics System. Multiple cause of death data, 1999–2018. https://wonder.cdc.gov/mcd.html.

